# Prevalence of the virulence genes and their correlation with carbapenem resistance amongst the *Pseudomonas aeruginosa* strains isolated from a tertiary hospital in China

**DOI:** 10.1007/s10482-023-01869-2

**Published:** 2023-10-17

**Authors:** Xiaohuan Wang, Kaijing Gao, Cuicui Chen, Cuiping Zhang, Chunmei Zhou, Yuanlin Song, Wei Guo

**Affiliations:** 1 Department of Laboratory Medicine, Zhongshan Hospital, Fudan University, 111 Yi Xue Yuan Road, Shanghai, 200032 People’s Republic of China; 2 Department of Pulmonary and Critical Care Medicine, Zhongshan Hospital, Fudan University, 180 Feng Lin Road, Shanghai, 200032 People’s Republic of China

**Keywords:** *Pseudomonas aeruginosa*, Type III secretion system, Carbapenem, Virulence, *exoS*, Genotype

## Abstract

*Pseudomonas aeruginosa* is one of the top-listed pathogens in nosocomial infection. It is notorious for its complicated virulence system and rapid adaptability to drugs or antimicrobials. In this study, we aimed to evaluate the prevalence of sixteen virulence genes in four groups including type III secretion system, biofilm formation, extracellular toxin biosynthesis and enzymes amongst 209 clinical *Pseudomonas aeruginosa* strains. We investigated the different distribution patterns of virulence genotypes based on carbapenem-resistant phenotype or the carriage of carbapenemase genes. The detection rate of each virulence gene varied greatly. *phzM* and *plcN* were detected in all collected strains, while *pilB* and *exoU* were only carried by a small portion of isolates (6.7% and 16.3%). Additionally, the number of genotypes observed in each group of examined virulence genes ranged from 4 to 8. Only the distribution of genotypes of type III secretion system showed statistical difference between carbapenem-mediated or carbapenem-resistant and carbapenem-sensitive strains. The virulence genotype of *Pseudomonas aeruginosa* was possibly interrelated to its resistance mechanism. Further research suggested that one particular TTSS genotype exhibited higher ratio in carbapenemase-producing strains and *exoS* was less frequently detected in CRPA strains carrying carbapenemase gene. Generally, the significant genetic diversity of virulence genes amongst *Pseudomonas aeruginosa* strains was highlighted in this study. Specific TTSS genotypes were associated with carbapenem-resistance. In particular, certain incompatibility might exist between *exoS* and carbapenemase genes, which provided valuable information for further understanding the relationship between carbapenem resistance and virulence.

**Supplementary Information:**

The online version contains supplementary material available at 10.1007/s10482-023-01869-2.

## Introduction

*Pseudomonas aeruginosa* (*P. aeruginosa*) is a common clinical gram-negative bacterium. As one of the top-listed pathogens in nosocomial infections, it is widely distributed on human skin, polluted medical devices and even disinfectants. *P. aeruginosa* is likely to cause acute or chronic multi-system (e.g., respiratory and urinary tract, bacteremia and soft tissues) infections among immunocompromised individuals (Qin et al. 2022). In addition, the characteristics of rapid adaptability to drugs or antimicrobials resistance and a complicated virulence system make it extremely difficult to treat effectively (Blomquist and Nix 2021).

*Pseudomonas aeruginosa* can combat the adverse host environment by producing a variety of virulence factors. Its virulence factors are composed of various substances which play an important role in the pathogenic process. Type III secretion system (TTSS) components, encoded by *exoU*, *exoS*, *exoT* and *exoY* genes, are reported to induce cytotoxic and invasive phenotypes (Galle et al. 2012; Horna and Ruiz 2021). It is one of the most extensively-studied virulence factors with increasing evidence for its predictive value of poor prognosis (Juan et al. 2017). Other extracellular virulence, such as toxins (e.g., toxin A and pyocyanin) and enzymes (e.g., elastases, proteases, phospholipases, and neuraminidases), also contribute to the pathogenicity of bacteria: aiding colonization, destroying protein structures and degrading many crucial proteins of the host cells (Ostroff et al. 1990; Toder et al. 1994; Iiyama et al. 2017). Furthermore, biofilm formation helps *P. aeruginosa* to survive in hypoxic or other harsh environments, and can lead to chronic infections that are difficult to eradicate (Maurice et al. 2018). Some biofilm matrix molecules (e.g., alginate) and cell appendages (e.g., type IV pili) are necessary for biofilm formation and are also considered as important virulence factors (Mann and Wozniak 2012). Those virulence factors are crucial for *P. aeruginosa* to cause infection. However, information about prevalence status of these factors amongst clinical isolates in China is inadequate.

In addition to the impact on the pathogenicity of *P. aeruginosa*, the correlation between virulence and drug resistance has also attracted extensive attention. Many studies had reported that the presence or expression of virulence genes was related to drug resistance. Some studies suggested that they are antagonistic, while other studies reported the resistant *P. aeruginosa* isolates with high virulence levels (Finlayson and Brown 2011; Jeannot et al. 2008; Pereira et al. 2015). *exoU*, one remarkable effector of TTSS, was reported to be related to fluoroquinolone resistance based on the results of a higher proportion of fluoroquinolone-resistance among *exoU*^+^ isolates (Wong-Beringer et al. 2008; Agnello and Wong-Beringer 2012). Therefore, genes determining virulence and related to drug resistance in *P. aeruginosa* clinical strains may form a specific combination type through appropriate regulation to better adapt to the hospital environment (Liao et al. 2022). It is meaningful to develop molecular epidemiology researches about virulence-genotype and resistance-phenotype to increase the understanding of *P. aeruginosa* infection at the molecular or genetic level.

Carbapenem-resistant *P. aeruginosa* (CRPA) is one of the major challenges in clinical practice, making it more difficult to treat patients (Yoon and Jeong 2021; Kucisec-Tepes 2004). Additionally, it was shown that some CRPA strains may clonally spread worldwide with specific dominant sequence types (Papagiannitsis et al. 2017). This study aimed to evaluate the prevalence of sixteen virulence genes of four virulence types mentioned above (TTSS: *exoU*, *exoS*, *exoT*, *exoY*; biofilm formation: *algD*, *pilA*, *pilB*; extracellular toxin biosynthesis: *toxA*, *phzM*, *phzS*; enzymes biosynthesis: *plcN*, *plcH*, *aprA*, *lasB*, *nan1* and *nan2*). We also compared the distribution difference of genotypes amongst subgroups of CRPA and non-CRPA strains. Moreover, this study also investigated whether the presence of particular virulence gene varied with respect to the carriage of carbapenemase gene in CRPA strains.

## Materials and methods

### Collection and identification of bacterial isolates

209 clinical isolates of *P. aeruginosa* were collected from 209 distinct clinical patients through eliminating duplicate strains in this study. And they were derived from the Microbiology Department of Laboratory Medicine in Zhongshan Hospital Affiliated to Fudan University in China. The verification of each isolate was based on the growth in blood agar plate at aerobic conditions and the identification was performed by utilizing MALDI-TOF/TOF (bioMérieux, Craponne, France) to single colony with VITEK® MS CHCA (bioMérieux, Craponne, France). MALDI-TOF/TOF achieved the precise microbial identification by its certain proteomic workflows and rigorous machine algorithms. Specifically, unknown bacteria dissolved in matrix solution of CHCA was detected to get the unique protein fingerprint and characteristic peaks which was assigned corresponding weights based on its frequency of occurrence amongst different bacterial strains. The obtained comprehensive information was then compared with the protein fingerprint inside the database. Accordingly, the final identification result and confidence were accurately identified.

### Antimicrobial susceptibility test

Antimicrobial susceptibility test of each isolate was detected by Vitek2 Compact system using GN 335 cards (bioMérieux, Craponne, France). It was a miniaturized and simplified version of automatic detection system using microdilution method to determine the minimum inhibitory concentration (MIC). This system was able to achieve automatic dissolution of drugs inside the card using prepared standard bacterial solution. Then, the bacteria growth within the specified time was monitored to obtain the MIC value of each drug. Results were interpreted according to standards established by the Clinical and Laboratory Standards Institute (CLSI. Performance standards for antimicrobial susceptibility testing.). CRPA isolates referred to *P. aeruginosa* strains that were mediated (MIC: 4 μg/mL) or resistant (MIC: ≥ 8 μg/mL) to imipenem and /or meropenem, Non-CRPA isolates referred to *P. aeruginosa* strains that were sensitive to imipenem and meropenem (MIC: ≤ 2 μg/mL).

### Bacterial genomic DNA isolation

Well-separated colonies of *P. aeruginosa* strains were cultured in fluid medium of Luria broth (LB) (Solarbio, Shanghai, China) at 37 °C overnight with constant shaking (200 rpm/min). Then, bacterial genomic DNA was isolated by employing the EasyPure® Bacteria Genomic DNA Kit (TransGen Biotech, Beijing, China) according to the manufacturer’s protocol. DNA samples were then stored at −20 °C before further use in PCR assay for carbapenemase and virulence genes detection.

### Carbapenemase and virulence genes detection

Carbapenemase genes (*bla*_KPC_, *bla*_GES_, *bla*_IMP_, *bla*_VIM_, *bla*_NDM_, *bla*_SIM_ and *bla*_OXA_) that have been reported in China were included in the present study for PCR detection. Sixteen virulence genes noted globally were also examined in this research. Briefly, whole-DNA extracts were detected using 2 × TransTaq®-T PCR SuperMix kit (TransGen Biotech, Beijing, China) under the following conditions: denaturation for 5 min at 94 °C, followed by 35 cycles of 94 °C for 30 s, 50–60 °C for 30 s, and 72 °C for 30 s, followed by a final extension step of 10 min at 72 °C. The primers involved in this study were listed in Table 1.

**Table 1:** The sequences of PCR primers applied in the present study and the expected sizes of amplification products

Gene detected	Primer	Primer sequence (5′ → 3′)	Product Size (bp)	References
*bla*_KPC_	F	CGTCTAGTTCTGCTGTCTTG	798	El-Mahdy and El-Kannishy (2019)
	R	CTTGTCATCCTTGTTAGGCG		
*bla*_GES_	F	ATGCGCTTCATTCACGCAC	864	Hayashi et al. (2021)
	R	CTATTTGTCCGTGCTCAGGA		
*bla*_IMP_	F	GGAATAGAGTGGCTTAAYTCTC	232	El-Mahdy and El-Kannishy (2019)
	R	GGTTTAAYAAAACAACCACC		
*bla*_VIM_	F	GATGGTGTTTGGTCGCATA	390	El-Mahdy and El-Kannishy (2019)
	R	CGAATGCGCAGCACCAG		
*bla*_NDM_	F	GGTTTGGCGATCTGGTTTTC	621	Poirel et al. (2011)
	R	CGGAATGGCTCATCACGATC		
*bla*_SIM_	F	TACAAGGGATTCGGCATCG	571	Queenan and Bush (2007)
	R	TAATGGCCTGTTCCCATGTG		
*exoU*	F	ATGCATATCCAATCGTTG	2000	Petit et al. (2013)
	R	TCATGTGAACTCCTTATT		
*exoS*	F	CTTGAAGGGACTCGACAAGG	504	Bogiel et al. (2021)
	R	TTCAGGTCCGCGTAGTGAAT		
*exoY*	F	CGGATTCTATGGCAGGGAGG	289	Bogiel et al. (2021)
	R	GCCCTTGATGCACTCGACCA		
*exoT*	F	AATCGCCGTCCAACTGCATGCG	152	Bogiel et al. (2021)
	R	TGTTCGCCGAGGTACTGCTC		
*algD*	F	ATGCGAATCAGCATCTTTGGT	1310	Bogiel et al. (2021)
	R	CTACCAGCAGATGCCCTCGGC		
*pilA*	F	ACAGCATCCAACTGAGCG	1675	Bogiel et al. (2021)
	R	TTGACTTCCTCCAGGCTG		
*pilB*	F	TCGAACTGATGATCGTGG	408	Bogiel et al. (2021)
	R	CTTTCGGAGTGAACATCG		
*toxA*	F	GGTAACCAGCTCAGCCACAT	352	Bogiel et al. (2021)
	R	TGATGTCCAGGTCATGCTTC		
*phzM*	F	ATGGAGAGCGGGATCGACAG	875	Bogiel et al. (2021)
	R	ATGCGGGTTTCCATCGGCAG		
*phzS*	F	TCGCCATGACCGATACGCTC	1752	Bogiel et al. (2021)
	R	ACAACCTGAGCCAGCCTTCC		
*plcN*	F	GTTATCGCAACCAGCCCTAC	466	Bogiel et al. (2020)
	R	AGGTCGAACACCTGGAACAC		
*nan2*	F	ACAACAACGGGGACGGTAT	1161	Bogiel et al. (2020)
	R	GTTTTGCTGATGCTGGTTCA		
*lasB*	F	GGAATGAACGAAGCGTTCTC	300	Bogiel et al. (2020)
	R	GGTCCAGTAGTAGCGGTTGG		
*nan1*	F	AGGATGAATACTTATTTTGAT	1316	Bogiel et al. (2020)
	R	TCACTAAATCCATCTCTGACCCGATA		
*aprA*	F	TGTCCAGCAATTCTCTTGC	1017	Bogiel et al. (2020)
	R	CGTTTTCCACGGTGACC		
*plcH*	F	GAAGCCATGGGCTACTTCAA	307	Bogiel et al. (2020)
	R	AGAGTGACGAGGAGCGGTAG		

PCR product was identified as previously described (Ghanem et al. 2023; Bogiel et al. 2023). It was separated by agarose gel electrophoresis in 1 × Tris–EDTA (TE) buffer. Nucleic Acid Gel Stain (Yeasen Biotechnology, Shanghai, China) achieved the final visual identification of separated bands using gel imaging system (Bio-Rad, Feldkirchen, Germany). *P. aeruginosa* strains carrying the particular genes and the PAO1 strain were served as positive or negative controls of PCR.

### Statistical analysis

All analyses were performed by the software of IBM SPSS Statistics 26.0 (SPPS Inc., Cary, NC). Approach of chi square test (χ^2^), Fisher-Freeman-Halton test and Criterion of *p* < 0.05 were used to determine the significance of the observed differences in virulence genes distribution amongst the tested strains, and with respect to their presence into two subgroups of distinct strains (Carbapenemase -positive and Carbapenemase -negative).

### Ethics statement

The ethical approval was obtained from the Ethics Committee at Zhongshan Hospital Affiliated to Fudan University (B2022-044R) (Approved on 25 February 2022). All clinical isolates used in this study were collected previously from routine microbiological specimens that were anonymized, and patients were not physically involved in this study.

## Results

### Clinical isolates

We collected 209 nonrepetitive clinical strains of *P. aeruginosa* that were comprised of 106 Carbapenem-resistant (CRPA) and 103 Carbapenem-sensitive strains (non-CRPA). As shown in Table S1, they were respectively isolated from respiratory tract (128/209, 61.3%), urine (24/209, 11.5%), drainage (22/209, 10.5%), pus (10/209, 4.8%), bile (9/209, 4.3%), secretion (7/209, 3.3%), hydrothorax and ascites (3, 1.4%), tissue (3/209, 1.4%), catheter (1/209, 0.5%) and other specimens (2/209, 1.0%). Additionally, genes of carbapenemase were positively detected in 20 CRPA strains (20/106, 18.9%).

### Prevalence of genes encoding virulence factors

**Fig. 1:**
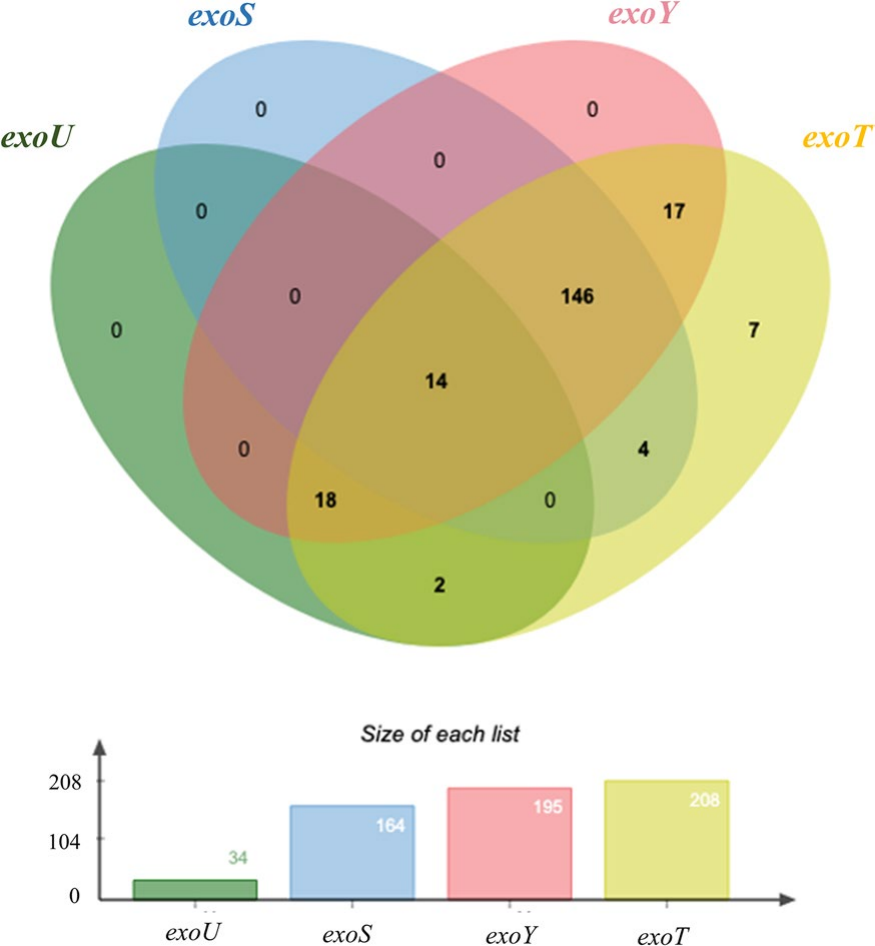
The prevalence and co-existence of TTSS genes amongst the tested clinical strains of *P. aeruginosa* (Venn diagram, n = 209)

Assessment of examined virulence factors in this study demonstrated a wide variety of gene prevalence. As shown in Fig. 1, the prevalence of four TTSS genes were as follows: *exoT* (99.52%) > *exoY* (93.30%) > *exoS* (78.47%) > *exoU* (16.27%). And there were fourteen strains carrying these four genes simultaneously.

**Fig. 2:**
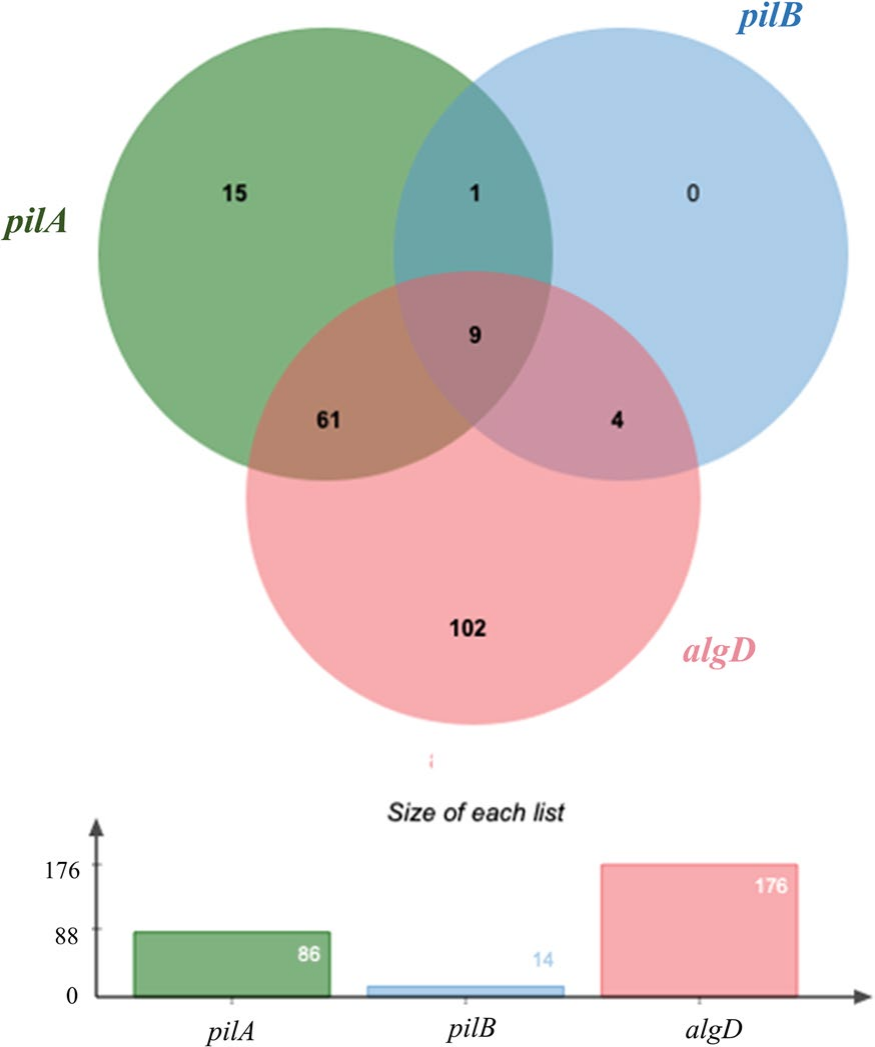
The prevalence and co-existence of genes related to biofilm biosynthesis amongst the tested strains of *P. aeruginosa* (Venn diagram, n = 209)

Of the three genes of virulence factors that were related to biofilm biosynthesis (Fig. 2), *algD* was detected with the highest frequency (84.2%), and *pilB* with the lowest frequency (6.7%) was the least rare in all examined genes. The detection frequency of *pilA* was 41.15%. The lone *algD* was observed in most of clinical isolates, while the number of strains carrying three genes above concurrently was only nine.

**Fig. 3:**
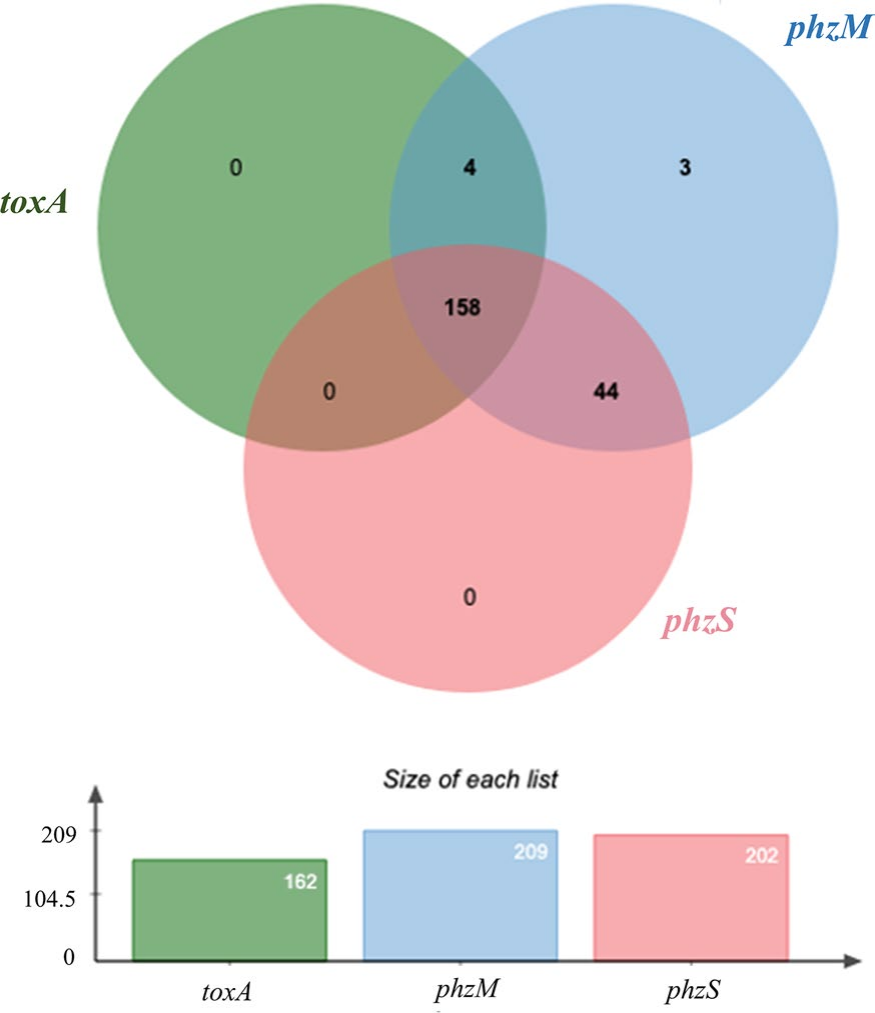
The prevalence and co-existence of genes related to the biosynthesis of toxic substances amongst the tested strains of *P. aeruginosa* (Venn diagram, n = 209)

The occurrence of the virulence genes involved in producing toxin A and pyocyanin were as follows: *toxA*-77.51%; *phzM*-100%; *phzS*-96.65% (Fig. 3). *phzM* was detected in all tested strains, and most of them (158) also carried genes of *phzS* and *toxA*.

**Fig. 4:**
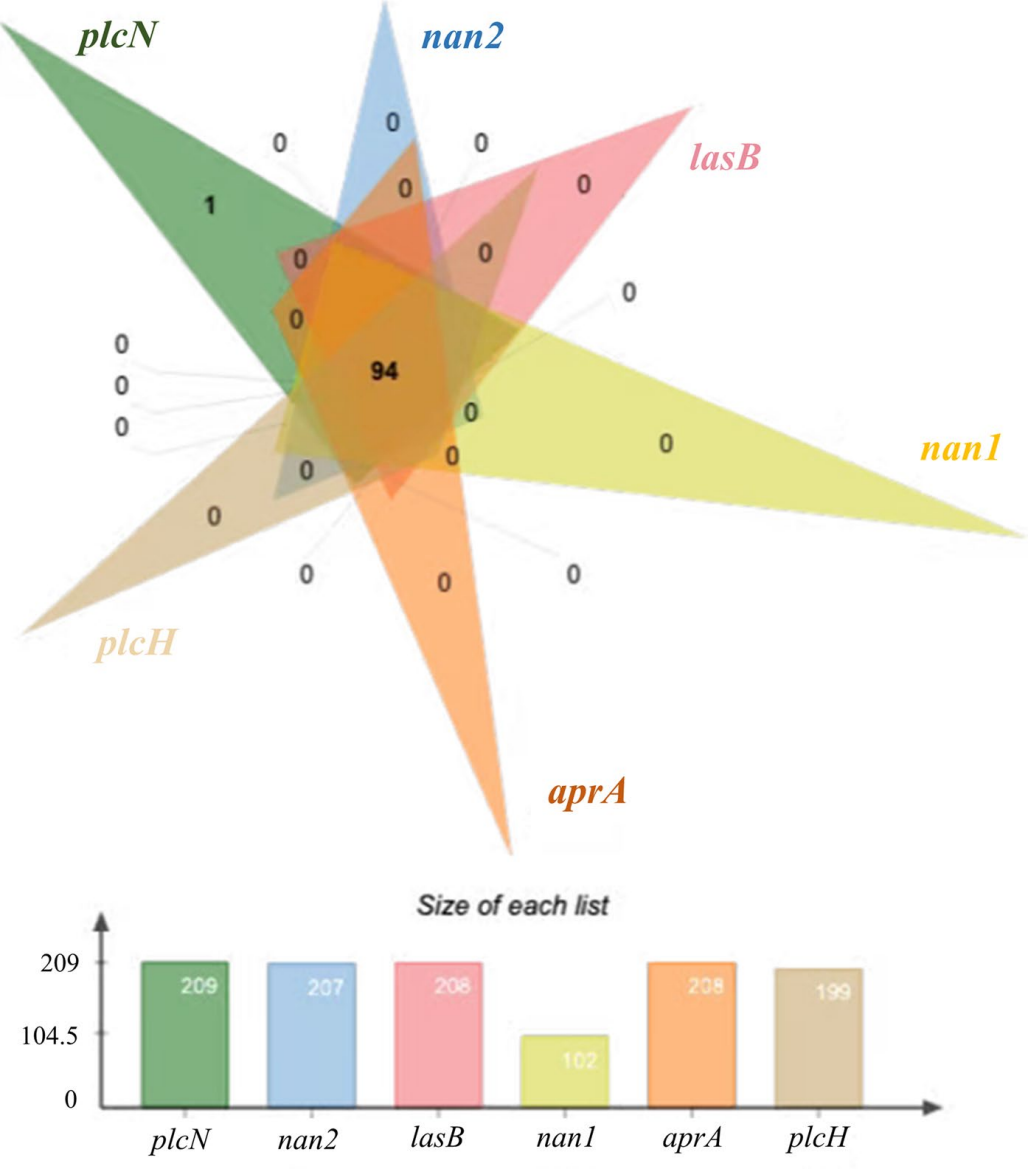
The prevalence and co-existence of genes related to enzyme production amongst the tested strains of *P. aeruginosa* (Venn diagram, n = 209)

Six enzyme-related virulence genes were detected with generally high frequency in tested strains of this study (Fig. 4). Among which, *plcN* was noted in all strains, and the detection frequencies of *aprA*, *lasB*, *nan2 plcH* and *nan1* were 99.5%, 99.5%, 99.0%, 95.2% and 48.8% respectively. Nearly half of the bacteria strains (94) possessed six genes together.

### Virulence genotype and their distributions

**Fig. 5:**
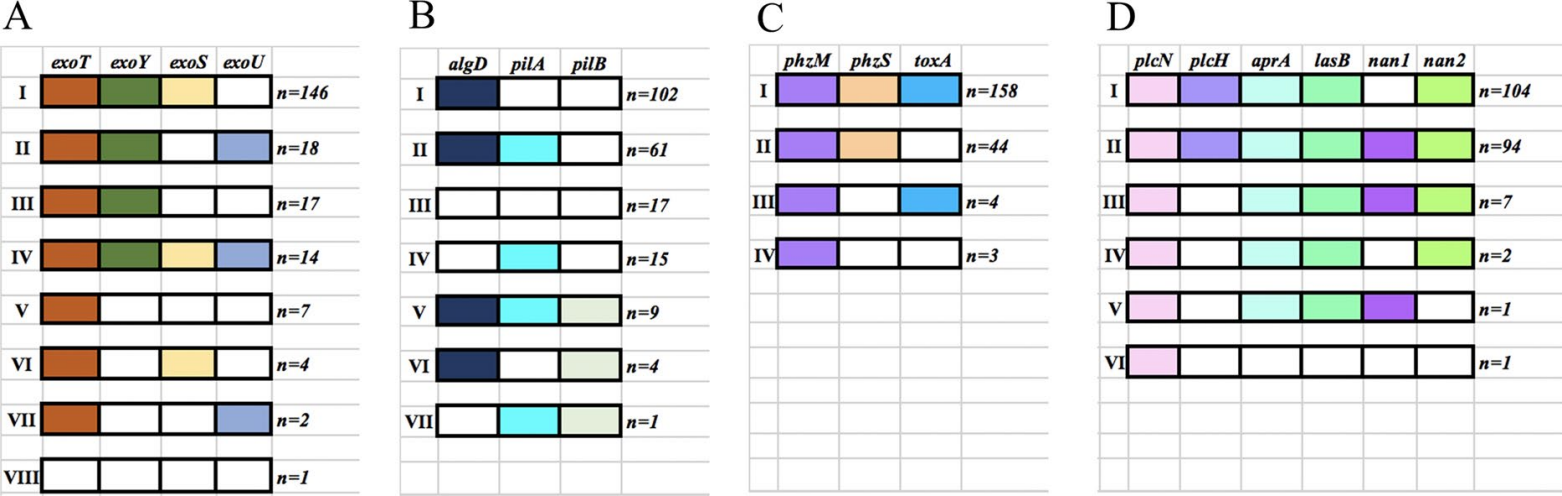
Genotypes of four groups of virulence genes detected amongst the 209 *P. aeruginosa* isolates. **a**, genotypes of TTSS genes amongst the 209 isolates; **b**, genotypes of genes related to biofilm biosynthesis amongst the 209 isolates; **c**, genotypes of genes related to the biosynthesis of toxic substances amongst the 209 isolates; **d**, genotypes of genes related to enzyme production amongst the 209 isolates

As shown in Fig. 5, various genotypes were separately observed in four groups of related virulence genes, and each group exhibited several scarce genotypes.

Then, a statistically significant difference (Fisher-Freeman-Halton test, *p* = 0.018) was observed in the overall distributions of TTSS genotypes between CRPA and non-CRPA isolates (Table 2). However, there was no statistical difference in the overall distribution of genotypes in other three groups, which were presented in Table S2, Table S3 and Table S4. Interestingly, TTSS genotype V (*exoT*^+^ *exoY*^−^ *exoS*^−^ *exoU*^−^) isolates were all gathered in CRPA strains although the number of bacterial strains was relatively small (100.0% vs 0.0%, *p* = 0.002). These results indicated that there might be a specific correlation between particular TTSS genotypes and the phenotype of carbapenem resistance.

**Table 2:** The distribution of TTSS genotypes amongst 209 strains of *P. aeruginosa* with respect to carbapenem-resistance

	TTSS genotypes							
	I	II	III	IV	V	VI	VII	VIII
CRPA (n = 106)	71	9	8	10	7	0	1	0
%	48.6%	50.0%	47.1%	71.0%	100.0%	0.0%	50.0%	0.0%
Non-CRPA (n = 103)	75	9	9	4	0	4	1	1
%	51.4%	50.0%	52.9%	29.0%	0.0%	100.0%	50.0%	100.0%

The carriage of carbapenemase genes by bacteria is one of the important mechanisms of resistance to carbapenems. To further understand the potential relevance between TTSS genotype and the presence of carbapenemase gene, the distribution of TTSS genotype amongst CRPA strains with carbapenemase gene (carbapenemase +) or not (carbapenemase -) was analysed (Table 3). Specifically, TTSS genotype II exhibited higher ratio in carbapenemase ( +) CRPA (25.0% vs. 4.7%, *p* = 0.011). Of four genes of TTSS, only *exoS* was absent in the genotype II, indicating that some certain incompatibility maybe existed between *exoS* and carbapenemase genes.

**Table 3:** The distribution of TTSS genotypes amongst the CRPA strains with respect to the carriage of carbapenemase genes

	TTSS genotypes							
	I	II	III	IV	V	VI	VII	VIII
Carbapenemase ( +) (n = 20)	10	5	2	1	2	0	0	0
%	50.0%	25.0%	10.0%	5.0%	10.0%	0.0%	0.0%	0.0%
Carbapenemase ( −) (n = 86)	61	4	6	9	5	0	1	0
%	70.9%	4.7%	7.0%	10.5%	5.8%	0.0%	1.2%	0.0%

### Distributions of individual gene of TTSS among CRPA strains

To further investigate the possible association of *exoS* and carbapenemase gene, we compared the frequency of individual gene of TTSS (*exoU*, *exoS*, *exoY* and *exoT*) between carbapenemase ( +) and carbapenemase (-) CRPA strains. As shown in Table 4, significant difference in the gene distribution between two subgroups of CRPA strains was observed for *exoS* (55.00% vs. 88.40%, *p* = 0.012). *exoS* was less frequently detected in CRPA strains of carbapenemase ( +), which further implied that it might possess a tendency to be incompatible with genes related to carbapenemase biosynthesis.

**Table 4:** The distribution of individual gene of TTSS amongst the CRPA strains with respect to the carriage of carbapenemase genes (statistical analysis was developed by chi square test)

	*exoU*	*exoS*	*exoY*	*exoT*
Carbapenemase ( +) (n = 20)	6	11	18	20
%	30.0%	55.0%	90.0%	100.0%
Carbapenemase (−) (n = 86)	14	70	80	86
%	16.3%	81.4%	93.0%	100.0%

## Discussion

*P. aeruginosa* is one of the most important pathogens worldwide characterized by high genetic. The vast majority of isolates possess virulence genes involved in the biosynthesis of pyocyanin, phospholipase C, alkaline protease, elastase B and some exoenzymes. Actually, the virulence trait of *P. aeruginosa* strain is a complex topic. Particular genotype composition might be one representative facet of their variable pathogenicity potential and sophisticated drug-resistance spectrum, most likely in a subgroup of strains.

The virulence of *P. aeruginosa* is jointly regulated by multiple signaling layers, and result in the multidimensional harmfulness to the host (Balasubramanian et al. 2013; Jimenez et al. 2012). This study focuses on the epidemiological distribution of important virulence factors related to TTSS, biofilm formation, extracellular toxin and destructive enzyme, and sixteen related genes were evaluated. Specifically, *phzM*, and *plcN* were detected in all collected clinical strains. *pilB* and *exoU* were only carried by a small portion of strains (6.7% and 16.3%), which is similar to but not entirely consistent with other relevant studies involving clinical isolates from blood or urine samples in Poland and Korea (Park and Koo 2022; Bogiel et al. 2022). Interestingly, one strain of our study carrying all of the sixteen virulence genes was detected. It was also an extensive-drug resistant (XDR) *P. aeruginosa* with resistance to six types of antibiotics, indicating that there was a possibility that the characteristics of significant drug resistance and high level of virulence could exist simultaneously in the complex medical environment. Besides, an overwhelming majority of *P. aeruginosa* strains were positively detected for the genes of *phzM* and *phzS* that were related to pyocyanin biosynthesis, suggesting that pyocyanin is one of the most common virulence determinants of this species. Fuse et al. had showed that multi-drug resistant (MDR) *P. aeruginosa* carrying genes coding metallo-β-lactamase (MBL) decreased the pyocyanin-producing ability (Fuse et al. 2013). It was noteworthy that in this study *phzM* and *phzS* were identified in only two CRPA isolates producing MBL simultaneously. Additionally, biofilm formation, one of the most common pathogenic factors for *P. aeruginosa* infection, increased the difficulty of removing the pathogen from the hospital environment and medical equipment (Thi et al. 2020). The detected proportions of related genes of *algD* (84.21%), *pilA* (41.15%) and *pilB* (6.70%) of this study were similar to results reported previously (Bogiel et al. 2022; Kamali et al. 2020; Stewart et al. 2011). Since genes encoding structural pilins of *P. aeruginosa* were detected at a relatively low frequency, alginate synthesis might be a more significant determinant with regard to the biofilm-associated virulence. In the present study, enzyme-related genes existed in almost all tested isolates except *nan1*, which was positively detected in about half of *P. aeruginosa* strains.

Many studies had tried to analyze the potential correlation between virulence and resistance spectrum of *P. aeruginosa* and got contradictory results. It was previously reported that *exoU*^+^ genotype of *P. aeruginosa* was positively correlated with moderate resistance phenotype and negatively correlated with XDR (Cabot et al. 2012; Peña et al. 2015), and *exoU*^+^ genotype was illustrated to be more likely to be resistant to fluoroquinolone with gyrA mutation and overexpression of efflux pump (Wong-Beringer et al. 2008; Agnello and Wong-Beringer 2012). In this study, eight TTSS genotypes of *P. aeruginosa* were found in 209 clinical isolates, including a rare genotype lacking all the four TTSS genes which was reported previously (Elsen et al. 2014). Additionally, genotypes of TTSS in this study were comprehensively classified based on *exoU*, *exoS*, *exoY* and *exoT* instead of merely considering *exoU* like other studies mentioned previously. And TTSS genotype V (*exoT*^+^ *exoY*^−^ *exoS*^−^ *exoU*^−^) of *P. aeruginosa* with a relatively low level of virulence gene were all resistant to carbapenem, which was likely to support the view of antagonism correlation between virulence and resistance. However, relevant studies were not enough to reveal the association between such genotype and drug resistant pattern. Further research with larger clinical sample size was worthwhile to investigate the association and potential mechanism.

Recently, with increased number of CRPA clinical isolates reported, the situation of carbapenem resistance has become even more severe. CRPA has been listed among the “critical” members of pathogens by WHO, nosocomial infection caused by CRPA and its clonal spread is one of the major global challenges of public health (Daikos et al. 2021; Mancuso et al. 2021). Researches about genetic features, mechanisms of carbapenem resistance and clinical prognosis of CRPA had been widely reported, but studies related to the prevalence of exhaustive virulence genes and their distributions in clinical strains are relatively inadequate. In the present study, the prevalence of the seven genes encoding carbapenemase (*bla*_KPC_, *bla*_GES_, *bla*_IMP_, *bla*_VIM_, *bla*_NDM_, *bla*_SIM_ and *bla*_OXA_) were also evaluated. The result showed that only 18.9% (20 of 106) of CRPA were positively detected, most of them were *bla*_KPC_ and *bla*_GES_ that belong to class A beta-lactamases which have been reported in China (Wang et al. 2006; Ge et al. 2011). Metallo-beta-lactamase (MBL), known as class B beta-lactamases, was most frequently reported in the published data from China (Fang et al. 2022; Feng et al. 2017), while only twol isolates of CRPA were detected that carry *bla*_NDM_ and *bla*_SIM_ respectively in this study. Since the studied strains are derived mainly from hospitalized patients in different provinces and cities, indicating that the prevalence of specific genes coding for carbapenemase could vary according to geographical distribution and potential endemic intrahospital spread. The epidemiological characteristic of carbapenemase gene was also reported to be linked to geographical location (Yoon and Jeong 2021). A study showed that the prevalence of carbapenemase gene amongst CRPA strains ranged from 0.0% to 44.3% in multiple global areas (Lee et al. 2022). In the present study, the presence of carbapenemase genes in the CRPA strains accounted for 18.9% of the resistance to carbapenems, which was consistent to the previous study (Zhang et al. 2015). The resistance mechanisms to carbapenems among *P. aeruginosa* have been found to be diverse. In addition to carrying carbapenemase genes, loss of outer membrane protein OprD and overexpression of efflux pump also contributed to carbapenems resistance (Farra et al. 2008; Masuda et al. 2000; Chalhoub et al. 2016). Therefore, other genetic traits of carbapenem resistance mechanisms mentioned above in the remaining isolates need further investigation.

In this study, we analyzed the different distribution of TTSS genotype based on carbapenem-resistant phenotype or the carriage of carbapenemase genes. It was found that *exoS* was inclined to reside in carbapenemase (-) CRPA strain, indicating that *exoS* might be incompatible with genes related to carbapenemase biosynthesis to some extent in clinical strains. We also investigated data from some published researches to analyze the distribution of exoS amongst CRPA strains of Carbapenemase ( +) or Carbapenemase (−) collected from several countries worldwide (Bogiel et al. 2021; Ferreira et al. 2015; Lee et al. 2013; Bellés et al. 2018), Interestingly, the results were consistent to our studies based on clinical isolates of *P. aeruginosa* from China (Table S5). Since *P. aeruginosa* had a complicated regulatory network controlling virulence and drug resistance, and the potential antagonism between *exoS* and carbapenemase gene might be beneficial for bacteria to obtain longer survival and infection (Liang et al. 2014). The finding of specific correlation between some virulence and carbapenemase genes is interesting and worthy of further investigation.

In conclusion, high genetic diversity of virulence was detected amongst clinical *P. aeruginosa* strains. Many clinical strains were found carrying virulence genes related to the biosynthesis of toxic substances and enzymes, while the prevalence of biofilm-related genes is generally low. Significantly, the distribution of TTSS genotypes showed statistical differences between the subgroup strains of CRPA and non-CRPA, implying the potential correlation between virulence and resistance. Moreover, Significant lower prevalence of *exoS* was noted in CRPA strains with respect to the presence of carbapenemase gene in this study. This suggested the existence of certain incompatibility between these two genes. It is worthy of further research with larger samples size and in-depth mechanism investigation in the future.

### Supplementary Information

Below is the link to the electronic supplementary material.

Supplementary file1 (DOCX 30 KB)

## Data Availability

Please contact author for data requests.

## Abbreviations

CRPA: Carbapenem-resistant *P. aeruginosa*
MBL: Metallo-beta-lactamase
MDR: Multi-drug resistant
TTSS: Type III secretion system
XDR: Extensive-drug resistant
