# Supplementary material for: Prevalence of the virulence genes and their correlation with carbapenem resistance amongst the Pseudomonas aeruginosa strains isolated from a tertiary hospital in China
Source: Antonie Van Leeuwenhoek. 2023 Oct 17;116(12):1395–406. doi: 10.1007/s10482-023-01869-2 (PMC10645663; doi:10.1007/s10482-023-01869-2)
Supplement: Supplementary file 1 — Supplementary file1 (DOCX 30 KB) [file 10482_2023_1869_MOESM1_ESM.docx]

**Prevalence of the virulence genes and their correlation with carbapenem resistance amongst the *Pseudomonas aeruginosa* strains isolated from a tertiary hospital in China**

Xiaohuan Wang^1†^, Kaijing Gao^2†^, Cuicui Chen^2^, Cuiping Zhang^2^, Chunmei Zhou^1^, Yuanlin Song^2*^, Wei Guo^1*^

^1^Department of Laboratory Medicine, Zhongshan Hospital, Fudan University, Shanghai, China. ^2^Department of Pulmonary and Critical Care Medicine, Zhongshan Hospital, Fudan University, Shanghai, China.

^†^ These authors have contributed equally to this work.

W.G. and Y.S. are co-corresponding authors

Professor Wei Guo, Department of Laboratory Medicine, Zhongshan Hospital, Fudan University, 111 Yi Xue Yuan Road, Shanghai 200032, P.R. China. E-mail: guo.wei@zs-hospital.sh.cn

Professor Yuanlin Song, Department of Pulmonary and Critical Care Medicine, Zhongshan Hospital, Fudan University, 180 Feng lin Road, Shanghai 200032, P.R. China. E-mail: song.yuanlin@zs-hospital.sh.cn

**Table S1.** Clinical strains of *P. aeruginosa* from different sample sources

| **Clinical strains** | **Sputum & BALF** | **Urine** | **Drainage** | **Pus** | **Bile** | **Secretion** | **Hydrothorax and ascites** | **Tissue** | **Other specimens** | **Catheter** | **Total** |
| --- | --- | --- | --- | --- | --- | --- | --- | --- | --- | --- | --- |
|  | n = 128 | n = 24 | n = 22 | n = 10 | n = 9 | n = 7 | n = 3 | n = 3 | n = 2 | n = 1 | n = 209 |
| CRPA | 65 | 13 | 11 | 5 | 6 | 1 | 2 | 0 | 2 | 1 | 106 |
| % | 61.3% | 12.3% | 10.4% | 4.7% | 5.7% | 1.0% | 1.8% | 0.0% | 1.8% | 1.0% |  |
| Non-CRPA | 63 | 11 | 11 | 5 | 3 | 6 | 1 | 3 | 0 | 0 | 103 |
| % | 61.1% | 10.7% | 10.7% | 4.9% | 2.9% | 5.8% | 1.0% | 2.9% | 0.0% | 0.0% |  |

**Table S2.** The genotype distribution of biofilm-related genes amongst the tested strains of *P. aeruginosa* (statistical analysis was developed by Fisher-Freeman-Halton test)

|  | **Genotype of biofilm-related genes** | | | | | | | |  |
| --- | --- | --- | --- | --- | --- | --- | --- | --- | --- |
|  | | I | II | III | IV | V | VI | VII | |
| CRPA (n) | | 52 | 23 | 12 | 10 | 5 | 3 | 1 | |
| % | | 51.0% | 37.7% | 70.6% | 66.7% | 55.6% | 75.0% | 100.0% | |
| Non-CRPA (n) | | 50 | 38 | 5 | 5 | 4 | 1 | 0 | |
| % | | 49.0% | 62.3% | 29.4% | 33.3% | 44.4% | 25.0% | 0.0% | |

**Table S3.** The genotype distribution of genes related to biosynthesis of toxic substances amongst the tested strains of *P. aeruginosa* (statistical analysis was developed by Fisher-Freeman-Halton test)

|  | **Genotype of genes related to biosynthesis of toxic substances** | | | | |  |
| --- | --- | --- | --- | --- | --- | --- |
|  | | I | II | III | IV | |
| CRPA (n) | | 82 | 22 | 0 | 2 | |
| % | | 51.90% | 50.00% | 0.00% | 66.70% | |
| Non-CRPA (n) | | 76 | 22 | 4 | 1 | |
| % | | 48.10% | 50.00% | 100.00% | 33.30% | |

**Table S4.** The genotype distribution of enzyme-related genes amongst the tested strains of *P. aeruginosa* (statistical analysis was developed by Fisher-Freeman-Halton test)

|  | **Genotype of enzyme-related gene** | | | | | | |  |
| --- | --- | --- | --- | --- | --- | --- | --- | --- |
|  | | I | II | III | IV | V | VI | |
| CRPA (n) | | 56 | 46 | 4 | 0 | 0 | 0 | |
| % | | 53.8% | 48.9% | 57.1% | 0.0% | 0.0% | 0.0% | |
| Non-CRPA (n) | | 48 | 48 | 3 | 2 | 1 | 1 | |
| % | | 46.2% | 51.1% | 42.9% | 100.0% | 100.0% | 100.0% | |

**Table S5.** Data related to the distribution of *exoS* amongst CRPA strains with carbapenemase genes positive or negtive (statistical analysis was developed by chi square test)

|  | This study | Research 1 | Research 2 | Research 3 | Research 4 | Total |
| --- | --- | --- | --- | --- | --- | --- |
| Carbapenemase (+) | 11(20) | 6(33) | 3(10) | 0(15) | 4(23) | 24(101) |
| *%* | 55.0% | 18.2% | 30.0% | 0.0% | 17.4% | 23.8% |
| Carbapenemase (-) | 70(86) | 57(74) | 53(75) | 29(42) | NA | 209(277) |
| *%* | 81.4% | 77.0% | 70.7% | 69.0% | NA | 75.5% |
| *P* | 0.012 | <0.001 | 0.028 | <0.001 | NA | <0.001 |
| References | / | 27 | 51 | 52 | 53 | / |
